# Quinine in Acute Rheumatism

**Published:** 1846-09

**Authors:** 


*Quinine in Acute Rheumatism.*—Three years have elapsed since M. Briquet communicated the success which attended the use of quinine in cases of acute articular rheumatism at the Hospital Cochin. Several practitioners have resorted to it with the same result: but its employment has become by no means general, partly in consequence of too large doses being given at first, and various accidents in consequence ensuing. M. Briquet formerly gave from 4 to 6 scruples in the 24 hours, but now gives but from 1 to 4, discontinuing it when any sign of prostration manifests itself. He employs the neutral salt rendered soluble by sulphuric acid. From the first or second night sleeplessness disappears, and a little later there is a more or less marked diminution of the pain and swelling of the joints. From the third to the sixth day the rheumatism may become cured; but when the cure is so prompt as this there is usually some return of pain, with or without swelling, again requiring the use of quinine. As a general rule the patients are cured or notably relieved from the ninth to the twelfth day of treatment.—*Med. Chir. Rev. from Gazette Medico-Chirurgicale.*

